# Resilience and Post-traumatic Stress Disorder in the Swiss Alpine Rescue Association

**DOI:** 10.3389/fpsyt.2022.780498

**Published:** 2022-03-23

**Authors:** Christian Mikutta, Julia J. Schmid, Ulrike Ehlert

**Affiliations:** ^1^University Hospital of Psychiatry and Psychotherapy, University of Bern, Bern, Switzerland; ^2^Privatklinik Meiringen, Meiringen, Switzerland; ^3^Department of Clinical Psychology, University of Zurich, Zurich, Switzerland

**Keywords:** mountain rescue, trauma, post-traumatic stress disorder, resilience, sense of coherence

## Abstract

**Objective:**

The present study aimed to assess the frequency of trauma exposure, the prevalence of possible post-traumatic stress disorder (PTSD), the extent of resilience, and sense of coherence among personnel of the Swiss alpine rescue association (ARS).

**Methods:**

Using a trilingual online survey approach, 465 mountain rescuers of the ARS were surveyed using the Posttraumatic Diagnostic Scale (PDS), the PTSD Checklist 5 (PCL-5), the Resilience Scale 13 and 14 (RS-13/-14), the Perceived Stress Scale 10 (PSS-10), the General Health Questionnaire 12 (GHQ-12), the Pittsburg Sleep Quality Index (PSQI), the Sense of Coherence Scale 13 (SOC-13), and the Berlin Social Support Scales (BSSS).

**Results:**

Although the rate of mountain rescuers having witnessed or experienced a traumatic event was high (71%), the prevalence of possible PTSD was low (0.9%). The sample showed high resilience and high sense of coherence. Resilience was positively correlated with work experience. Low perceived stress and high sense of coherence predicted resilience. The severity of PTSD symptoms was mainly predicted by low sense of coherence. Sense of coherence mediated the interaction between resilience and severity of PTSD symptoms.

**Conclusion:**

The findings suggest that resilience and sense of coherence are indicative for the low prevalence of possible PTSD among mountain rescuers, and may therefore represent valuable screening and training parameters for mountain rescue personnel.

## Introduction

A vast number of studies have highlighted the increased risk of experiencing traumatic events among emergency and rescue personnel (1), pertaining not only to events experienced as a victim, but also those experienced as a witness (2). Indeed, studies show that between 80 and 100% of emergency and rescue personnel have experienced at least one traumatic event during service (3–5). Due to this accumulation of traumatic events, emergency and rescue personnel experience higher levels of psychological stress and are consequently at greater risk of developing mental health problems (6, 7).

Meta-analyses and systematic reviews have demonstrated that the point prevalence of PTSD in emergency and rescue personnel lies at ~10% (1, 8–10), as compared to 1.3–3.5% in the general population (1, 10, 11). However, prevalence rates vary broadly across different studies, ranging from 0 to 46% (9). Moreover, emergency and rescue personnel show an increased prevalence of comorbid psychiatric symptoms such as depression, substance abuse, and social dysfunction (12).

In recent years, the scientific focus has shifted more toward health-promoting factors (13), and the concept of resilience has received increased attention as a protective factor in this regard (14). While there is no generally accepted definition of resilience, the concept might best be described as the process of adapting well in the face of adversity, trauma, tragedy, threats, or significant sources of stress (15). Surprisingly, studies analyzing resilience directly in emergency and rescue personnel are lacking, although the few studies comparing resilience between rescue personnel and the general population reported higher resilience among rescue personnel (16).

To date, almost all studies exploring PTSD symptoms or health-promoting factors in this group have focused on professional rescue personnel, while data on voluntary rescue and emergency personnel are sparse (17). A recent study found that seasonal firefighters had a higher risk of developing PTSD than did professional firefighters (18).

In Switzerland, the alpine rescue service (ARS) is a voluntary organization. Mountain rescuers receive training consisting of first aid and rescue techniques for the alpine environment for summer and winter, with additional options for specialized training (e.g., specialist for helicopter rescues in alpine environment, rescue dog handler).

Research has shown that traumatic events like avalanches, rockfalls, or falls while climbing are frequent in alpine environments (19). Indeed, in 2020, the ARS received a total of 999 emergency calls and assisted 1,319 people during their missions (20).

The present study aimed to assess (a) the frequency and types of traumatic situations during mountain rescue missions and (b) the frequency of possible PTSD among mountain rescuers. Moreover, we sought to examine (c) the extent of resilience among mountain rescuers as compared to the general population using the samples of Leppert et al. (21), Wagnild and Young (22), and Callegari et al. (23), and (d) differences between mountain rescuers with and without possible PTSD. Finally, (e) we evaluated predictors of resilience and severity of PTSD symptoms.

## Methods

### Procedure

The recruitment was carried out in cooperation with the management of the Swiss alpine rescue service, who sent an e-mail to all ARS members in November 2020. In Switzerland, 63% of the population is German-speaking, 22% is French-speaking, and 8% is Italian-speaking. Therefore, the survey was trilingual, with an option to choose the language at the beginning of the survey. Besides information on the planned study, the mail contained a link and a QR code to the online platform of the University of Zurich. Reminder mails were sent on December 1st 2020 and January 13th 2021. The recruitment process was completed by the end of January 2021. The Ethics Committee of the Faculty of Arts and Humanities, University of Zurich approved the study.

### Participants

In 2020, a total of 2,330 mountain rescuers were registered with the ARS. Of these, 73% (1,708) were German-speaking, 14% (295) French-speaking, and 13% (292) Italian-speaking. Thirteen percent of the rescuers were female (292) and 87% (2,038) were male.

A total of 456 mountain rescuers took part in the online survey, resulting in a response rate of 20%. Of the participants, 72% (334) were German-speaking, 13% (24) were French-speaking, and 15% (25) were Italian-speaking. The sample consisted of 6.5% (26) female and 93% (434) male participants. The mean age was 45.6 years (±11.4). Table 1 describes the sociodemographic characteristics of the mountain rescuers.

**Table 1:** Sociodemographic characteristics of the Swiss mountain rescuers (*N* = 465).

	**Total sample** ***N*** **=** **465**	
	***N*** **(%)**	***M*** **(*****SD*****)**
Language		
German	334 (71.8%)	
French	62 (13.3%)	
Italian	69 (14.8%)	
Sex		
Female	30 (6.5%)	
Male	434 (93.3%)	
Age		45.6 (11.4)
Civil status		
Single	40 (8.6%)	
In a relationship	95 (20.4%)	
Married	293 (63.0%)	
Registered relationship	6 (1.3%)	
Divorced	31 (6.7%)	
Education		
Special school	10 (2.2%)	
Secondary school	40 (8.6%)	
Apprenticeship	174 (37.4%)	
Grammar school	23 (4.9%)	
University	137 (29.5%)	
Other	80 (17.2%)	

### Measures

The online assessment covered demographic (sex, age, civil status, education) and psychosocial variables. To assess the type of potential traumatic events, a modified version of the Posttraumatic Diagnostic Scale (PDS) was used, similar to the scale employed in the aforementioned study by Sommer and Ehlert (19). The scale differentiates between events witnessed and events experienced and includes traumatic events that are encountered in an alpine environment.

To estimate PTSD symptoms according to the DSM-5, we administered the PTSD Checklist for DSM-5 (PCL-5) (27–29). We chose the PCL-5, since it is the most commonly used self-rating instrument and covers the DSM-V criteria (30). This self-rating instrument consists of 20 items rated on a 5-point scale. The overall score ranges from 0 to 80 and depicts the severity of PTSD symptoms, while the cut-off score (32/80) distinguishes between participants with or without possible PTSD (27). The cut-off was chosen based on the recommendation of the U.S. Department of Veterans Affairs (31). Since the diagnosis of a self-rating instrument is not verified, we termed participants meeting the cut-off as having possible PTSD. When referring to the total score of the PCL-5, we use the term severity of PTSD symptoms. Subsyndromal PTSD was defined as meeting the diagnostic criteria for Cluster B and Cluster C or Cluster B and Cluster E (32).

To measure resilience, we used the Resilience Scale 13 and 14 (RS-13/-14) (21–23). The German version of the scale consists of 13 items while the Italian and French versions comprise the original 14 items. The statements are rated on a 7-point Likert scale, and the overall score (range 14–98, German version: 13–91) reflects the extent of resilience. We chose the RS-13/14 since it covers the five core characteristics of resilience (purpose, perseverance, self-reliance, equanimity, and authenticity) (22). For being able to compare the RS 13 and 14 scores from the different language groups we used the mean value of each participant (total score of each participant divided by 13 or 14).

Perceived stress was assessed using the short version of the Perceived Stress Scale 10 (PSS-10) (33–36), with 10 items rated on a 5-point scale (range 10–50). The PSS is a widely used self-rating instrument, with a general nature of questions, which makes this questionnaire suitable for general populations (37).

To detect participants with diagnosable psychiatric disorders, we used the short version of the General Health Questionnaire (GHQ), the GHQ-12 (26, 38–40). The scale consists of 12 items rated on a 4-point Likert scale. Similar to Sommer and Ehlert, the scoring method (0011) and the threshold score of 4/5 were used for case identification (screening use) and the Likert scoring method (0123) was used for total and subscale scores (survey use) (range 0–36). This questionnaire is a widely used scale in general practice studies of physical illness and distress and has been recommended for screening and interviewing trauma victims (39).

We assessed quality of sleep using the Pittsburg Sleep Quality Index (PSQI) (41–44). The PSQI consists of seven items rated on a 3-point scale, resulting in an overall score ranging from 0 to 21, with higher scores reflecting a lower quality of sleep.

To examine participants' sense of coherence, we used the short version of the sense of coherence scale 29, the sense of coherence scale 13 (SOC-13) (45–48). The SOC-13 consists of 13 items rated on a 7-point Likert scale. Only the overall score (range 13–91) was used in the present study. This instrument showed to be cross-cultural valid and reliable (49).

Finally, we used the Berlin Social Support Scales (BSSS) to estimate the perceived social support (50). For the present study, we only used the scales Perceived Emotional Social Support and Perceived Instrumental Social Support, resulting in eight items rated on a 4-point Likert scale (range 8–32). Since there is no Italian version available, the scales were translated into Italian by a highly skilled Italian speaker with a medical background and were validated by a second Italian speaker.

### Statistical Analyses

All statistical analyses were conducted using SPSS version 25. Data were tested for normal distribution using a Shapiro-Wilk test and for homoscedasticity using a Levine test.

First, to estimate the number of traumatic incidents and the percentage of mountain rescuers who had experienced/witnessed one traumatic event, we used the corresponding items of the PDS.

Second, to estimate the percentage of participants showing a possible PTSD, we used the cut-off score of the PCL-5.

Third, we computed a Spearman correlation between work experience and resilience (RS-13/14). Additionally, we computed the mean resilience scores of the sample using the RS-13/14 and conducted a one-factor ANOVA (using quartiles of work experience as group variable) in order to estimate group differences in resilience and work experience.

Fourth, we compared selected variables (RS-13/14, PSS-10, GHQ-12, PSQI, SOC-13, BSSS) in participants with at least subsyndromal PTSD and the remainder of the sample, using a Mann-Whitney *U*-test. *P*-values were Bonferroni-corrected to a significance level of *p* = 0.0083 (0.05/6).

Fifth, to define reliable predictors for the severity of PTSD symptoms (as measured by the total score of the PCL-5) and resilience, we computed Spearman's rank correlation and biserial correlations with the number of traumatic events (0, ≥1), number of different traumatic events (as a victim and a witness), work experience, number of missions, professional training, age, perceived stress (PSS-10), general mental health (GHQ-12), quality of sleep (PSQI), sense of coherence (SOC-13), and perceived social support (BSSS). Variables with significant results (*p* < 0.05) were used as input and PCL-5 and RS13/14 as output for a hierarchical multiple regression models. The models were bootstrapped (1,000 iterations, bias-corrected, acceleration method). Finally, to estimate the mediation effect, we performed a post hoc PROCESS macro V. 3.5.2 (bootstrapping 5,000 iterations, heteroscedasticity-consistent standard error estimators) as implemented in SPSS (51).

## Results

### Frequencies and Types of Trauma Exposure

**Figure 1:**
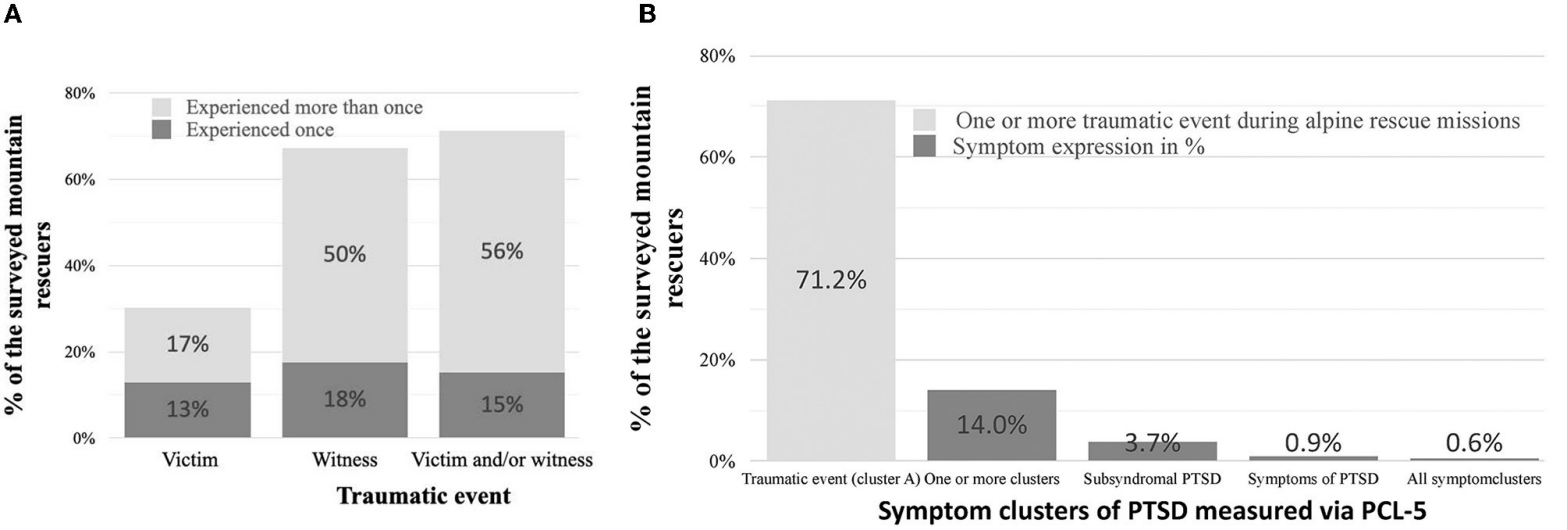
**(A)** Depicts the percentage of mountain rescuers who experienced a traumatic event either as victim, as witness or as victim/witness combined. **(B)** Depicts the prevalence of the clusters of PTSD.

In general, the personnel of the Swiss mountain rescue service had been exposed to many traumatic events. As victims, 12.9% had experienced one traumatic event and 17.4% had experienced more than one event. As witnesses, 17.6% had experienced one traumatic event and 49.7% had experienced more than one event. Accordingly, 15.1% had experienced one traumatic event as a victim or witness and 56.1% had experienced more than one traumatic event as a victim or witness. The most common events were falling while climbing (14.0% as a victim, 47.1% as a witness), avalanches (14.4% as a victim, 33.8% as a witness), and ice/rock falls (9.9% as a victim, 14.8% as a witness). Table 2 summarizes the frequencies and types of traumatic events. Figure 1A depicts the overall number of traumatic events.

**Table 2:** Traumatic events experienced by Swiss mountain rescuers (*N* = 465).

	**Sample** ***N*** **=** **465**		
	**Victim and witness** ***N* (%)**	**Victim** ***N* (%)**	**Witness** ***N* (%)**
Traumatic event during an ARS mission	331 (71.2%)	141 (30.3%)	313 (67.3%)
Once	70 (15.1%)	60 (12.9%)	82 (17.6%)
More than once	261 (56.1%)	81 (17.4%)	231 (49.7%)
Fall (e.g., crevasses, sports climbing)		65 (14.0%)	219 (47.1%)
Helicoptercrash		4 (0.9%)	17 (3.7%)
Avalanche		67 (14.4%)	157 (33.8%)
Ice- or rockfalls		46 (9.9%)	69 (14.8%)
Hypothermia or Frostbite		7 (1.5%)	44 (9.5%)
Snow blindness		1 (0.2%)	3 (0.6%)
Heatstroke		0 (0.0%)	6 (1.3%)
Lightening		11 (2.4%)	17 (3.7%)
Acute pulmonary or cerebral oedema		4 (0.9%)	19 (4.1%)
Exhaustion		5 (1.1%)	47 (10.1%)
Heart attack		4 (0.9%)	95 (20.4%)
Drowning (effective or potential)		18 (3.9%)	46 (9.9%)
Poisoning (e.g., CO_2_)		0 (0.0%)	15 (3.2%)
Hypoglykaemia		3 (0.6%)	21 (4.5%)
Fever		–	27 (5.8%)
Rescue death		28 (6.0%)	73 (15.7%)
Number of different traumatic events [*M* (*SD; R*)]			
Unexpected confrontation with body parts			248 (53.3%)
Once			78 (16.8%)
More than once			170 (36.6%)

### Post-traumatic Stress Symptoms

Only four of the 465 participants (0.9%) showed a possible PTSD, as defined by the cut-off score (32/80) on the PCL-5. Fourteen participants (2.8%) showed subsyndromal PTSD and 65 participants (14%) showed at least one symptom cluster. Figure 1B depicts the prevalences of the different symptom clusters. Table 3 provides detailed data on the symptom clusters.

**Table 3:** Psychological assessment of Swiss mountain rescuers (*N* = 465).

	**Sample** ***N*** **=** **465**		
	***N* (%)**	***M* (*SD; R*)**	**Max. range**
PCL-5		3.8 (5.7; 0–39)	0–80
Above Cut-off	4 (0.9%)		
Symptomcluster B	38 (8.2%)	1.0 (1.9; 0–15)	0–20
Symptomcluster C	21 (4.5%)	0.4 (1.0; 0–8)	0–8
Symptomcluster D	12 (2.6%)	1.0 (2.1; 0–15)	0–28
Symptomcluster E	29 (6.2%)	1.3 (2.1; 0–14)	0–24
All Clusters	3 (0.6%)		
Subsyndromal PTBS	17 (3.7%)		
RS-13/RS-14		5.8 (0.7; 1.3–7.0)	1–7
RS-13 Overall score		74.8 (10.0; 17–91)	13–91
RS-14 Overall score		81.0 (9.5; 36–96)	14–98
PSS-10		21.0 (4.8; 10–39)	10–50
GHQ-12 (Likert-method)		8.4 (3.5; 0–23)	0–36
Screening Cut-off	77 (16.6%)		
PSQI		3.5 (2.3; 0–14)	0–21
<6 (good sleeper)	401 (86.2%)		
6–10 (bad sleeper)	56 (12.0%)		
>10 (possible sleeping disorder)	8 (1.7%)		
SOC-13		74.8 (8.7; 34–91)	13–91
BSSS			
Overall score perceived social support		29.4 (3.3; 8–32)	8–32
Emotional perceived support		14.7 (1.8; 4–16)	4–16
Instrumental social support		14.6 (1.8; 4–16)	4–16

### Extent of Resilience

The German-speaking mountain rescuers scored on average 74.8 points (SD: 10.0, *n* = 334) on the RS-13 scale. The French- and Italian-speaking mountain rescuers scored on average 81.0 points (SD = 9.5, *n* = 131) on the RS-14.

### Interaction Between Resilience and Work Experience

A correlation emerged between resilience and work experience (r_s_ = 0.16, *p* = 0.001, two-tailed). In addition, we divided the sample into four quartiles according to their work experience in years. The one-factor ANOVA of the four quartiles yielded a significant difference [F_(3, 457)_ = 3.83, *p* = 0.010]: The mountain rescuers with a maximum of six years of work experience (lowest quartile, *n* = 107, M = 5.6, SD = 0.7) were significantly less resilient than those with more than 23 years of work experience (highest quartile, *n* = 119, M = 5.9, SD = 0.7, *post-hoc t*-test *p* = 0.008).

### Comparison Between Participants With and Without at Least Subsyndromal PTSD

Participants with at least subsyndromal PTSD showed significantly lower resilience (z = −4.27, *p* < 0.001, *r* =0.20), a significantly higher stress level (*z* = 3.57, *p* < 0.001, *r* = 0.17), poorer general mental health (*z* = 4.13, *p* < 0.001, *r* = 0.19), poorer quality of sleep (*z* = 4.41, *p* < 0.001, *r* = 0.20), lower sense of coherence (*z* = −4.78, *p* < 0.001, *r* = 0.22), and a lower level of perceived social support (*z* = −3.41, *p* < 0.001, *r* = 0.16) than those without subsyndromal PTSD.

### Predictors of the Severity of PTSD Symptoms

Significant negative Spearman's rank correlations emerged between overall PCL-5 score (indicating the severity of PTSD symptoms) and resilience (*r*_*s*_ = −0.16, *p* < 0.001, one-tailed, *N* = 465), sense of coherence (*r*_s_ = −0.33, *p* < 0.001, one-tailed, *N* = 465), perceived social support (*r*_s_ = −0.24, *p* < 0.001, one-tailed, *N* = 465), and age (*r*_s_ = −0.10, *p* = 0.031, two-tailed, *N* = 464).

Further we found a negative Spearman's rank correlations between overall PCL-5 score and general mental health (as measured by the GHQ) (*r*_s_ = 0.35, *p* < 0.001, one-tailed, *N* = 465). Please note that therefore more severe PTSD symptoms were associated with poorer mental health.

Similar, we found a positive Spearman's rank correlation between overall PCL-5 score and quality of sleep (*r*_s_ = 0.33, *p* < 0.001, one-tailed, *N* = 465), indicating that more sever PTSD symptoms were associated with poor sleep quality.

Moreover, we found significant positive Spearman's rank correlation between PCL-5 overall score (indicating the severity of PTSD symptoms) and perceived stress (*r*_s_ = 0.32, *p* < 0.001, one-tailed, *N* = 465), and number of different traumatic events as a victim (*r*_s_ = 0.16, *p* < 0.001, one-tailed, *N* = 465) and as a witness (*r*_s_ = 0.14, *p* = 0.001, one-tailed, *N* = 465).

No significant correlations emerged for number of traumatic events, number of missions, and work experience (all *p* > 0.05).

**Figure 2:**
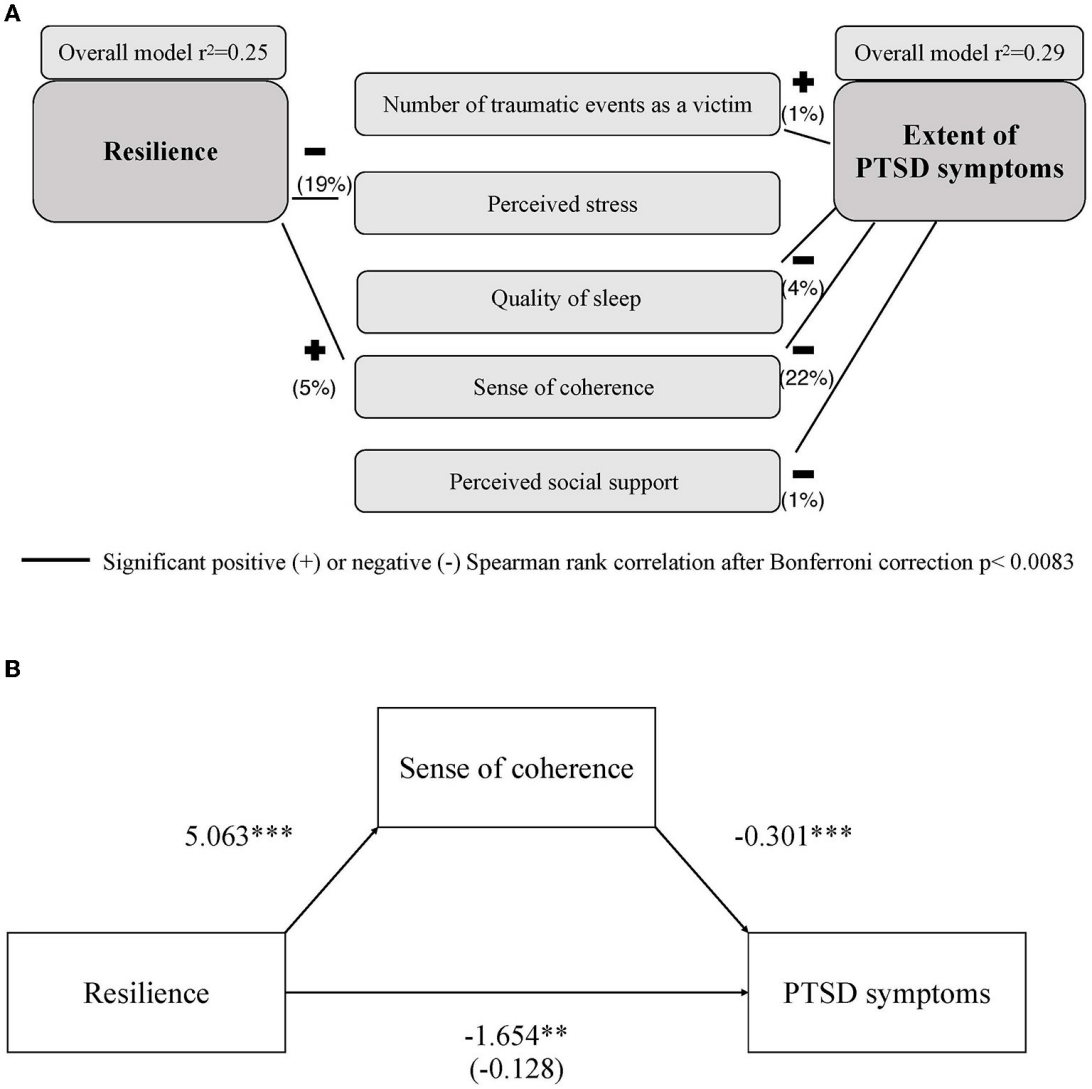
**(A)** Depicts the predictive model of resilience (left) and severity of PTSD symptoms (right). Black lines indicate the positive and negative Spearman rank correlations of resilience and severity of PTSD symptoms with the variables included in the respective models. Numbers in brackets indicate the explained variance of the individual predictive factors. **(B)** Shows the mediation model of sense of coherence (mediator) between resilience (predictor) and severity of PTSD symptoms (dependent variable). **p < 0.01, ***p < 0.001.

The aforementioned significant variables were subsequently used as input factors for a hierarchical regression model with PCL-5 overall score as output variable [*F*_(9, 454)_ = 21.93, *p* < 0.001, corrected *R*^2^ = 0.29, *N* = 464] and resulted in an explained variance of 29%. Sense of coherence was the strongest predictor (β = −0.30, *p* < 0.001, 22%), followed by quality of sleep (β = 0.17, *p* < 0.001, 4%), perceived social support (β = −0.12, *p* = 0.007, 1%), and number of different traumatic events as a victim (β = 0.10, *p* = 0.027, 1%) (Figure 2, upper panel right side).

### Predictors of Resilience

We found significant positive Spearman's rank correlations between resilience and, sense of coherence (*r*_s_ = 0.48, *p* < 0.001, one-tailed, *N* = 465), perceived social support (*r*_s_ = 0.29, *p* < 0.001, one-tailed, *N* = 465), number of traumatic events (*r*_pb_ = 0.09, *p* = 0.045, two-tailed, *N* = 465), number of different traumatic events as a witness (*r*_s_ = 0.12, *p* = 0.010, two-tailed, *N* = 465), work experience with the ARS (*r*_s_ = 0.16, *p* = 0.001, two-tailed, *N* = 461), and number of missions (*r*_s_ = 0.19, *p* < 0.001, two-tailed, *N* = 465). Further, we found a significant positive Spearman's rank correlation between resilience and general mental health (as measured by the GHQ) (*r*_s_ = −0.39, *p* < 0.001, one-tailed, *N* = 465). Therefore, as resilience increases, mental health improves. Similar, we found a significant negative Spearman's rank correlation between resilience and quality of sleep (*r*_s_ = −0.26, *p* < .001, one-tailed, *N* = 465) indicating that a higher resilience goes along with a better sleep quality.

Furthermore, we found negative correlations of resilience with the severity of PTSD symptoms (*r*_s_ = −0.16, *p* < 0.001, one-tailed, *N* = 465) and perceived stress (*r*_s_ = −0.46, *p* < 0.001, one-tailed, *N* = 465).

No significant correlation emerged for age and the number of traumatic events as a victim (all *p* > 0.05).

The aforementioned significant variables were subsequently used as input factors for a hierarchical regression model with resilience as output variable. The overall model emerged as significant [*F*_(11, 448)_ = 14.88, *p* < 0.001, corrected *R*^2^ = 0.25, *N* = 460) and resulted in an explained variance of 25%. The most reliable predictor was perceived stress (β = −0.24, *p* < 0.001, 19% explained variance), followed by sense of coherence (β = 0.23, *p* < 0.001, 5% explained variance) (Figure 2, upper panle left side).

### Mediation Analysis

A significant relationship was found between resilience (predictor) and severity of PTSD symptoms (dependent variable) (*B* = −1.65, *p* = 0.002). Resilience had a significant effect on sense of coherence (mediator) (*B* = 5.06, *p* < 0.001), and sense of coherence had a significant effect on the severity of PTSD symptoms (*B* = −0.30, *p* < 0.001). The effect of resilience on the severity of PTSD symptoms disappeared when sense of coherence was included in the model (*B* = −0.13, *p* = 0.729). Accordingly, the relationship between resilience and severity of PTSD symptoms was moderated by sense of coherence [indirect effect= −1.53, 95%-KI = (−2.29, −0.91)] (see Figure 2, lower panel).

## Discussion

The main findings of our study indicate that although a high number of mountain rescuers had experienced traumatic events, only a very small number met the cut-off for a possible PTSD. Moreover, we found high resilience scores within the sample of mountain rescuers. The most reliable predictor of the severity of PTSD symptoms was low sense of coherence, followed by poor quality of sleep, a low level of perceived social support, and a high number of different traumatic events as a victim. Further results indicated that low perceived stress and high sense of coherence were the strongest predictors of resilience in our sample.

The number of traumatic events experienced by the mountain rescuers was much higher than in the general population, with 71% of our sample having experienced a traumatic event during their missions. Nevertheless, this proportion was lower than in other studies covering emergency and rescue personnel (prevalences between 80 and 100%) (5, 52–55). The most important difference between our sample and the samples investigated in previous studies appears to be that the ARS is a voluntary organization and therefore has a lower rate of missions per person. Moreover, there is evidence that this subgroup has a higher risk of developing mental disorders due to greater structural barriers to the treatment of psychopathological consequences of traumatic events. The ARS has about 800–900 missions each year, resulting in an average of 3.4 missions per mountain rescuer annually. However, the numbers of missions per rescuer varied greatly across the sample, with 10% of the rescuers carrying out ~60% of the missions. Although the actual percentage of traumatic events is slightly lower than that reported for professional rescue workers, the number of traumatic events is still high. As such, the low prevalence of possible PTSD (1%) and subsyndromal PTSD (3%) is all the more surprising.

Although the present findings, along with the findings from previous studies (2, 56), revealed a rather low prevalence of possible PTSD in relation to the high levels of exposure to traumatic events, the prevalence of 1% found in the present ARS sample is far lower than in previous studies covering emergency and rescue personnel (~10%) (57) and even compared to the prevalence in the general population (1.3–3.5%) (1). In detail, Streb et al. (16) found a PTSD prevalence of ~4.3 % among paramedics, and Misra et al. (58) reported a prevalence of around 4% among the rescue personnel who responded to the 2005 London bombings. However, these two samples differ from the sample investigated in the current study, since they consisted of professional rescue workers. Moreover, Misra et al. (58) examined the prevalence following a single traumatic event. Our results are in contrast to existing research covering voluntary rescue and emergency personnel, which reported a slightly higher PTSD prevalence compared to professional rescue personnel (59–61).

A previous study on PTSD symptoms caused by mountain accidents revealed that between 14 and 31% of victims still showed different symptoms of PTSD 9 months after rock climbing accidents (62). Another study reported a PTSD symptom prevalence of 28% among mountaineers suffering an avalanche accident and of 43% among fully buried avalanche victims (63).

A possible explanation for the comparably lower prevalence of possible PTSD in our sample is the lower frequency of missions as compared to professional rescue personnel. Moreover, the ARS personnel seem to be exposed to fewer different types of traumatic events compared to other professional rescue personnel (64, 65). A recent study found that a larger number of different traumatic events increases the risk of developing PTSD symptoms (66). A further explanation might lie in the higher percentage of indirect traumatic events (about 60%) compared to direct traumatic events (30%), as indirect exposure has been associated with a lower risk of developing PTSD symptoms than direct exposure (2). A study by Sommer and Ehlert (19), which examined PTSD symptoms in Swiss mountain guides, found that the emotional reaction to traumatic events was low, pointing at a high tolerance of objectively dangerous situations.

Resilience among the mountain rescuers of our sample was found to be higher than in all three comparison samples representing the general population (21–23). This corresponds to recent evidence indicating higher resilience among German paramedics compared to the general population (16). An Italian study also found a high level of resilience in emergency personnel, and identified self-efficacy, collective efficacy, and sense of community as the main protective factors for mental health (67). Since our study provides only single time point data, we are unable to draw definitive conclusions on the etiology of the high resilience found in our ARS sample. One possible explanation lies in self-selection, insofar as individuals with higher resilience may be more likely to join the ARS in the first place and to remain with the ARS after joining. As another potential explanation, it may be that the level of work experience influences resilience, as our data suggest that mountain rescuers with less work experience (<7 years) show less resilience than those with more work experience (>23 years). This is in line with the findings of Gayton and Lovell (68), who reported that resilience in ambulance paramedics increased with time in service. In general, our findings support the line of research pointing to resilience as a dynamic factor rather than a personal trait (69).

The present data indicate that mountain rescuers meeting the criteria for a atleast subsyndromal PTSD differ from those without at least subsyndromal PTSD with regard to certain variables. Mountain rescuers with subsyndromal PTSD were significantly less resilient, showed poorer general mental health, lower sense of coherence, lower perceived social support, worse quality of sleep, and more perceived stress. These findings demonstrate that even subsyndromal PTSD results in detectable changes in mental wellbeing. This corresponds to previous studies that reported an association of subsyndromal PTSD with a lower level of functioning (70–72) as well as impairments in social and work-related matters, resulting in a diminished quality of life (30, 73). However, since effect sizes in the present study were small (*r* < 0.3) and the two group sizes differed considerably, we focused on predictors of severity of PTSD symptoms rather than on group differences.

To find predictive factors for the severity of PTSD symptoms in emergency and rescue personnel, it might be helpful to create screening programs for these individuals. In our study, lower sense of coherence, lower perceived social support, poorer quality of sleep, and a higher number of different traumatic events experienced as a victim emerged as the most reliable predictors of the severity of PTSD symptoms in a model that explained 29% of the variance. Within this model, sense of coherence was the strongest predictor (22% explained variance). This finding supports the results of a recent meta-analysis, which reported that sense of coherence explained 17% of the variance in PTSD symptoms after a traumatic event (74). Furthermore, studies by Streb and Schäfer (16, 24) likewise found that sense of coherence was the strongest predictor of PTSD symptoms in German paramedic personnel and other emergency and rescue personnel. In contrast, in the aforementioned study in Swiss mountain guides by Sommer and Ehlert (19), high sense of coherence was found. However, although sense of coherence was a significant predictor, it only explained 1% of the variance of the severity of PTSD symptoms. The authors concluded from this finding that sense of coherence is a factor of mental health rather than a protective factor against PTSD symptoms (19). Contrary to this assumption, Ragger (75) characterized sense of coherence as a protective factor against PTSD symptoms and additionally demonstrated that sense of coherence might positively influence the course of PTSD symptoms. Quality of sleep, perceived social support, and number of different traumatic events as a victim explained only 4, 1, and 1% of the variance, respectively.

Our data revealed perceived stress and sense of coherence to be the most reliable predictors of resilience in a model explaining 25% of the variance. Low perceived stress was the strongest predictor, explaining 19% of the variance, indicating that mountain rescuers who perceived a high level of stress were less resilient. There is evidence that more resilient emergency and rescue personnel experience less stress or are more capable of coping with challenging situations (76, 77). Moreover, it has been proposed that specific training programs to promote resilience might help emergency and rescue personnel to better cope with stress (76). Research also indicates that high resilience protects the individual from developing PTSD at high stress levels (78). In this regard, methods to increase resilience have also been developed and validated specifically for emergency and rescue personnel (79, 80).

In the present study, we found that sense of coherence mediated the association between the severity of PTSD symptoms and resilience, an effect that was also reported by Streb et al. (16) in German paramedic personnel. Moreover, Schnell also identified sense of coherence as a valid predictor of the severity of PTSD symptoms in voluntary firefighters (17). These findings indicate that sense of coherence might be a valuable target for improving resilience as well as for protecting against PTSD symptoms. Research indicates that specific programs are capable of optimizing sense of coherence (25). Indeed, even psychoeducation of emergency and rescue personnel is suitable for elevating sense of coherence (16). Furthermore, a study in Austrian ambulance personnel found that fostering sense of coherence is able to not only preserve general mental health but also improve it (75).

In sum, our results underline the importance of sense of coherence in the field of PTSD psychopathology. Specifically, sense of coherence proved to be a good predictor of PTSD symptoms and mediated the interaction between PTSD symptoms and resilience. Research indicates that sense of coherence might be enhanced by professional interventions, and might therefore be a reasonable target for training and education of rescue personnel (81).

The main limitation of our study is the rather low response rate of 20%, which might have led to systematic error in the sample. One reason for this low response rate might be that more than 50% of the contacted mountain rescuers had not undergone a mission within the last 12 months and were therefore not motivated to take part in the survey. Nevertheless, our sample proved to have captured the more active part (defined as having served at least in one mission in the last year) of the ARS (48% overall vs. 80% test sample). This considerably reduces the risk of underrepresenting traumatic events and consequently PTSD symptoms in the sample. Furthermore, since only self-report questionnaires were used, there is a risk of response bias. Finally, we did not include a scale for social desirability. Although evidence suggests that the tendency for socially desirable responding is strongly reduced in anonymous online surveys, bias in terms of social desirability cannot be definitively ruled out (82). Further research should include clinical interviews and longitudinal designs to address hypotheses regarding causality.

Our results suggest that despite a high prevalence of traumatic events, only a minority of mountain rescuers are burdened with mental health problems or a possible PTSD. We identified sense of coherence as an important predictive factor for the severity of PTSD symptoms and as an effective mediator of the interaction between the severity of PTSD symptoms and resilience. As such, sense of coherence might be a valuable variable for screening and training mountain rescue personnel.

## Data Availability Statement

The raw data supporting the conclusions of this article will be made available by the authors, without undue reservation.

## Ethics Statement

The studies involving human participants were reviewed and approved by the Ethics Committee of the Faculty of Arts and Humanities, University of Zurich approved the study. The patients/participants provided their written informed consent to participate in this study.

## Author Contributions

CM, JS, and UE designed the study. JS conducted the data acquisition and analyzed the data. CM wrote first draft of the manuscript, had full access to all of the data in the study, and took responsibility for the integrity of the data and the accuracy of the data analysis. All authors discussed the findings and edited the final manuscript.

## Conflict of Interest

The authors declare that the research was conducted in the absence of any commercial or financial relationships that could be construed as a potential conflict of interest.

## Publisher's Note

All claims expressed in this article are solely those of the authors and do not necessarily represent those of their affiliated organizations, or those of the publisher, the editors and the reviewers. Any product that may be evaluated in this article, or claim that may be made by its manufacturer, is not guaranteed or endorsed by the publisher.
